# Increasing intensities of *Anisakis simplex* third-stage larvae (L3) in Atlantic salmon of coastal waters of Scotland

**DOI:** 10.1186/s13071-020-3942-5

**Published:** 2020-02-12

**Authors:** Alexander J. Kent, Campbell C. Pert, Robert A. Briers, Karen Diele, Sonja Rueckert

**Affiliations:** 1 School of Applied Sciences, Edinburgh Napier University, Edinburgh, EH11 4BN Scotland; 2 Animal, Rural and Environmental Sciences, Nottingham Trent University, Southwell, NG25 0QF UK; 3 St Abbs Marine Station, The Harbour, St Abbs, Berwickshire, TD14 5PW UK

**Keywords:** Ascaridoid nematodes, Parasites, Red Vent Syndrome, *Salmo salar*, Stable isotopes

## Abstract

**Background:**

Red Vent Syndrome (RVS), a haemorrhagic inflammation of the vent region in Atlantic salmon, is associated with high abundance of *Anisakis simplex* (*s.s*.) third-stage larvae (L3) in the vent region. Despite evidence suggesting that increasing *A. simplex* (*s.s.*) intensity is a causative factor in RVS aetiology, the definitive cause remains unclear.

**Methods:**

A total of 117 Atlantic salmon were sampled from commercial fisheries on the East, West, and North coasts of Scotland and examined for ascaridoid parasites. Genetic identification of a subsample of *Anisakis* larvae was performed using the internal transcribed spacer (ITS) region of ribosomal DNA. To assess the extent of differentiation of feeding grounds and dietary composition, stable isotope analysis of carbon and nitrogen was carried out on Atlantic salmon muscle tissue.

**Results:**

In the present study, the obtained ITS rDNA sequences matched *A. simplex* (*s.s.*) sequences deposited in GenBank at 99–100%. Not all isolated larvae (*n* = 30,406) were genetically identified. Therefore, the morphotype found in this study is referred to as *A. simplex* (*sensu lato*). *Anisakis simplex* (*s.l.*) was the most prevalent (100%) nematode with the highest mean intensity (259.9 ± 197.3), in comparison to *Hysterothylacium aduncum* (66.7%, 6.4 ± 10.2) and *Pseudoterranova decipiens* (*s.l.*) (14.5%, 1.4 ± 0.6). The mean intensity of *A. simplex* (*s.l.*) represents a four-fold increase compared to published data (63.6 ± 31.9) from salmon captured in Scotland in 2009. Significant positive correlations between *A. simplex* (*s.l.*) larvae intensities from the body and the vent suggest that they play a role in the emergence of RVS. The lack of a significant variation in stable isotope ratios of Atlantic salmon indicates that diet or feeding ground are not driving regional differences in *A. simplex* (*s.l.*) intensities.

**Conclusions:**

This paper presents the most recent survey for ascaridoid parasites of wild Atlantic salmon from three coastal regions in Scotland. A significant rise in *A. simplex* (*s.l.*) intensity could potentially increase both natural mortality rates of Atlantic salmon and possible risks for salmon consumers due to the known zoonotic role of *A. simplex* (*s.s.*) and *A. pegreffii* within the *A. simplex* (*s.l.*) species complex.

## Background

Ascaridoid nematodes of the genus *Anisakis* Dujardin, 1845 colonise the digestive system of marine vertebrates [1]. Until recently, the nomenclature of species belonging to the genus *Anisakis* has been controversial [2]. The use of nuclear genetic markers, however, has revealed the existence of reproductive isolation between various sympatric and allopatric populations [1]. As a result, there are four clades of sibling species within the genus *Anisakis* that have been widely accepted. Clade 1, also known as the *A. simplex* (*sensu lato*) complex, consists of *A. berlandi* Mattiucci, Cipriani, Webb, Paoletti, Marcer, Bellisario, Gibson & Nascetti, 2014, *A. pegreffii* Campana-Rouget & Biocca, 1955, and *A. simplex* (Rudolphi, 1809) (*sensu stricto*). Clade 2 is formed by *A. ziphidarum* Paggi, Nascetti, Webb, Mattiucci, Cianchi & Bullini, 1998 and *A. nascettii* Mattiucci, Paoletti & Webb, 2009. Clade 3 is comprised of *A. physeteris* (Baylis, 1923), *A. brevispiculata* Dollfus, 1966 and *A. paggiae* Mattiucci, Nascetti, Dailey, Webb, Barros, Cianchi & Bullini, 2005, and Clade 4 contains *A. typica* (Diesing, 1860) Baylis 1920 [2]. Further genotypes *Anisakis* sp. 1 and *Anisakis* sp. 2 have been recognised and these show phylogenetic similarities to *A. typica* and *A. physeteris*, respectively; however, additional molecular genetic analyses are needed to fully clarify their taxonomic status [2].

*Anisakis simplex* (*s.s.*) is an Arctic Boreal species with a circumpolar distribution from approximately 35°N to the Arctic Seas [1]. Its heteroxenic life-cycle commonly involves crustaceans as first intermediate hosts, fish and squid species as second intermediate/paratenic hosts, and cetaceans as definitive hosts [1]. To date, at the adult stage *A. simplex* (*s.s.*) is found in 12 species of dolphins, porpoises, and whales and, as third-stage larvae (L3) in 50 pelagic, benthopelagic and demersal teleost fishes and four squid species [1].

The Atlantic salmon, *Salmo salar* (L.), is a second intermediate host of *A. simplex* (*s.s.*) (L3) larvae. In addition to the musculature, visceral organs and tissues in the body cavity such as the gut, pyloric caeca, liver and mesenteries, are the most common sites of infection [3]. Following reports of a condition which, in 2005, became known as Red Vent Syndrome (RVS) [4], large numbers of un-encapsulated *A. simplex* (*s.l.*) L3 were observed in gross lesions around the urogenital papilla region of Atlantic salmon, also referred to as the vent [5]. Subsequently, *A. simplex* (*s.l.*) larvae have been genetically assigned to *A. simplex* (*s.s.*) [5, 6]. Characterised by bleeding, swollen, and haemorrhagic vents [5], RVS has subsequently been recorded throughout populations of Atlantic salmon in the North Atlantic [6–8]. The ‘hyper-infestation’ of the vent region by *A. simplex* (*s.s.*) had not been previously recorded in Atlantic salmon or any other fish host of anisakid larvae [5] and has been suggested to be a result of increasing intensities of *A. simplex* (*s.s.*) in Atlantic salmon [9]. Additionally, the presence of L3 in the vent has been demonstrated to increase the likelihood of RVS symptoms [8].

*Anisakis simplex* (*s.s.*) intensity within a host is dependent on a number of biotic and abiotic factors at different geographical scales [10]. More specifically, the distribution of L3 and adult stages is generally shaped through biotic factors involved in transmission pathways, such as trophic interrelations between definitive, intermediate and transport hosts and their respective migrating behaviours [11].

Rising sea surface temperatures (SST) between 0.5–1.5 °C since 1901 [12] have been recorded throughout the Atlantic salmon’s natural range including common foraging areas of European populations [13]. Subsequent large-scale northward shifts of other intermediate hosts, e.g. warm water copepods [14], and an increase in the occurrence of warm water cetacean species in Scotland [15] have the potential to significantly affect *A. simplex* (*s.s.*) abundance and introduce other *Anisakis* species in coastal waters around Scotland through their roles in transmission, and reproductive capacity of *Anisakis*, respectively. Furthermore, with Atlantic salmon being an opportunistic feeder [16], differences in food availability and food web structure in common feeding grounds can have direct influences on levels of parasite abundance [8, 17].

To date, the definitive cause of the infection of the vent region by *A. simplex* (*s.s.*), and for the exhibition of RVS symptoms is unclear. Moreover, the differences in the migratory routes and feeding grounds of Atlantic salmon populations during their marine phase, and their influence on *A. simplex* (*s.s.*) intensity, remain cryptic. This study therefore, aimed to (i) assess the current intensity of *A. simplex* (*s.l.*) in Atlantic salmon of Scotland; (ii) investigate the relationship between *A. simplex* (*s.l.*) intensity in different body parts of Atlantic salmon in relation to the vent and to test the ‘hyper-infestation’ hypothesis proposed by Senos et al. [9]; (iii) explore any geographic differences in *A. simplex* (*s.l.*) abundance between coastal regions of Scotland; and (iv) investigate whether stable isotope ratios, as an indicator of feeding ground and dietary composition, are uniform between Atlantic salmon sampled from different coastal waters.

## Methods

### Study area and fish sampling

**Fig. 1:**
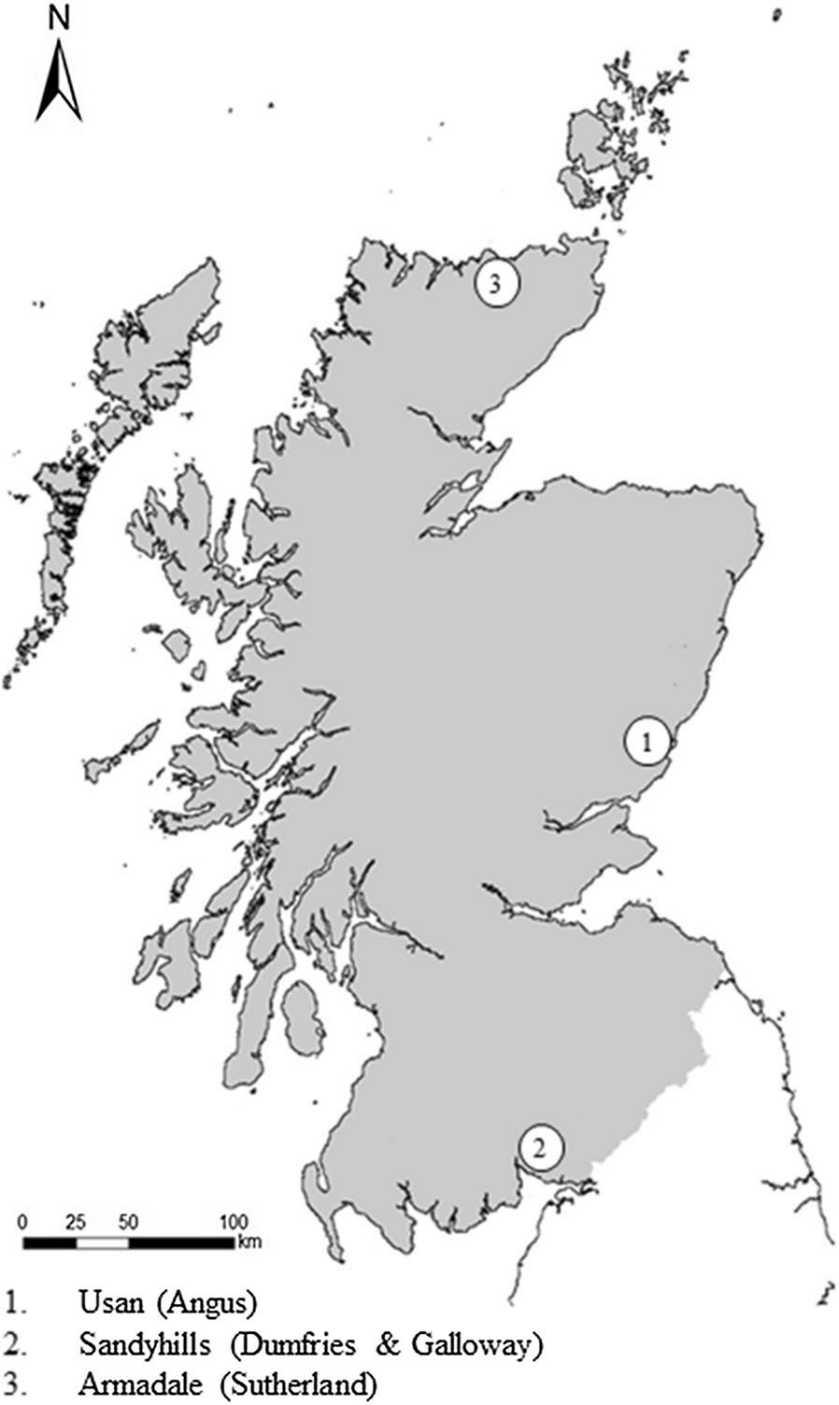
Sampling locations in Scotland

In total, 117 specimens of Atlantic salmon were examined for ascaridoid parasites. The fish were obtained from commercial inshore net fisheries on the East (*n* = 57), West (*n* = 34) and North (*n* = 26) coasts of Scotland between June-September 2015 (Fig. 1). Fish samples consisted of 72 male and 45 female specimens, with fork length and total weight ranging between 46.5–82.0 cm (mean ± standard deviation (SD): 57.6 ± 5.5 cm) and 0.75–5.25 (mean ± SD: 1.85 ± 0.68 kg), respectively. Individuals were sampled haphazardly in relation to the severity of RVS symptoms. Severity was determined following the external observation guidelines provided by the Fisheries Research Services (now Marine Scotland) [18]. Samples consisted of Atlantic salmon showing no (*n* = 19 East, *n* = 3 North and *n* = 4 West), mild (*n* = 14 East, *n* = 3 North and *n* = 4 West), moderate (*n* = 15 East, *n* = 6 North, and *n* = 13 West), and severe (*n* = 9 East, *n* = 14 North, and *n* = 13 West) RVS symptoms. Atlantic salmon not exhibiting RVS symptoms were selectively chosen from sample sites for comparative purposes. Several dorsal scales were removed from each fish and examined to assess fish age following the procedure provided by Smolyar & Bromage [19]. Fish were individually labelled, bagged and transported in polystyrene boxes with ice before being frozen at − 20 °C. Samples were frozen within 24 h to ensure no larval migration between tissues occurred [20].

### Parasitological examination

Atlantic salmon were defrosted overnight at 4 °C. Once thawed, each fish was weighed (total fish weight, TFW), measured (fork length, FL: anterior tip of the fish to the fork of the caudal fin) and photographed. A block of tissue (*c.*2 cm^3^) around the vent and urogenital papilla was excised and weighed prior to larvae removal (vent weight, VW). The body cavity was accessed through an incision made from the anus anteriorly towards the operculum. Visceral organs were removed, and each fish was weighed (gutted weight, GW). Visceral tissue weight (VTW) was calculated as the difference between TFW and GW. Ascaridoid parasites were recovered from the body cavity following visual examination. Muscle tissue was filleted and subsequently weighed (muscle weight, MW). Nematode larvae were removed from muscle tissue and visceral organs using the UV-press method of larval inspection as described by Karl & Leinemann [21] and later updated and modified by Karl & Levsen [22]. Nematode larvae were removed from all tissues and fixed in Davidsons’ AFA fixative solution for 24 h before being stored in glycerol-alcohol. Larvae were morphologically identified to discriminate between individuals of the genera *Anisakis, Pseudoterranova* (Nematoda: Anisakidae) and *Hysterothylacium* (Nematoda: Raphidascarididae) based on Berland [23], and keys from Arai & Smith [24].

### Enzymatic digestion

A random subsample of 11 whole Atlantic salmon carcasses was retained after the dissection and examination protocol was carried out, and each placed separately in labelled bags for enzymatic digestion to assess the accuracy of the parasitological examination described above. The subsample of Atlantic salmon was recorded to be exhibiting no RVS symptoms (*n* = 2 East), mild (*n* = 3 East and *n* = 1 North), moderate (*n* = 1 North and *n* = 1 West), and severe (*n* = 1 North and *n* = 2 West) symptoms. Carcasses were removed from the freezer ~12 h before digestion. The enzymatic digestion followed the method described by Jackson et al. [25], which was updated and modified by Noguera et al. [3].

### Genetic identification of *Anisakis* larvae

During parasitological examination, *Anisakis* larvae (*n* = 4) were collected from the body cavity (*n* = 2) and vent region (*n* = 2) of two Atlantic salmon (*n* = 1 East and *n* = 1 West) exhibiting severe RVS symptoms. *Anisakis* larvae were subsequently washed with physiological saline (Sigma-Aldrich, Irvine, UK) and then stored in 100% molecular grade ethanol (Thermo Fisher Scientific, Loughborough, UK) for molecular analysis. Each larva was cut into two pieces and homogenised using the TissueLyser LT (Qiagen, Manchester, UK) for 3 minutes at 50 Hz in 180 µl of tissue lysis buffer ALT (Qiagen). The solution was subsequently mixed with 20 µl of Proteinase K solution (20 mg/ml) (Qiagen) and incubated at 56 °C for 2 h. Total DNA was extracted using the DNeasy® extraction kit (Qiagen) according to the manufacturer’s instructions. The entire nuclear internal transcribed spacer (ITS) region (ITS1, 5.8S rDNA gene and ITS2) of the nuclear ribosomal DNA (rDNA) was amplified using the primers NC5 (5′-GTA GGT GAA CCT GCG GAA GGA TCA TT-3′) and NC2 (5′-TTA GTT TCT TTT CCT CCG CT-3′) according to Zhu et al. [26]. The PCR conditions followed those described by Zhu et al. [27] and the resultant PCR products were purified using UltraClean™ 15 DNA Purification kit (MO Bio, Carlsbad, California). Purified PCR products were premixed with 1 µl of NC5 or NC2 primers at a working concentration of 10 pM/µl and diluted to a concentration of 5 ng/µl prior to submission to Eurofins Genomics (Wolverhampton, UK) for sequencing. The four obtained sequences were deposited in the GenBank database under the accession numbers MN313576-MN313579.

The obtained sequences were compared with sequences retrieved from GenBank: *A. simplex* (*s.s.*) (AY826723), *Anisakis* sp. (AY821740 and AY821749), *A. berlandi* (JX535519), *A. pegreffii* (AB196671), *A. typica* (JQ912690), *A. brevispiculata* (JQ912694), *A. paggiae* (EU624345), *A. physeteris* (JQ912693), *A. nascettii* (JQ912692), *A. ziphidarum* (AY826725), *Ascaris suum* Goeze, 1782 (AB110023 and FJ418786), *Toxocara canis* Werner, 1782 (AJ002435 and FJ418788). Sequences for *A. suum* and *T. canis* were combined for each species to span the entire ITS region as described in previous studies [28]. A dataset of 15 sequences was aligned using ClustalW [29] and manually fine-tuned using MEGA v6 software [30] resulting in 855 unambiguously aligned sites. A pairwise distance calculation based on Kimura’s 2-parameter model [31] was performed on a dataset of 635 nucleotides (excluding gaps and missing data), and 921 nucleotides (including gaps and missing data) using MEGA v6 with bootstrap searches performed using 1000 pseudoreplicates.

### Stable isotope analysis

Dorsal muscle tissue (0.5 cm^3^) was excised from each fish sample and subsequently oven dried at 60 °C for 48 h. Dried samples were ground with a pestle and mortar to a fine, uniform powder. Between 0.6–0.8 mg of each sample was weighed on a Sartorius ENTRIS124-1S balance (Sartorius, Göttingen, Germany), placed in pressed tin capsules (5 × 3.5 mm) (Elemental Microanalysis, Okehampton, UK) and stored in a glass desiccator. Lipids were removed from a sub-sample of 45 ground dorsal muscle tissue as described by Folch et al. [32]. Sub-samples were subsequently re-dried and re-ground.

All samples were combusted in an Elementar Pyrocube elemental analyser connected *via* continuous flow to a Thermo Fisher Scientific - Delta Plus X mass spectrometer at the Scottish Universities Environmental Research Centre (SUERC) (East Kilbride, Glasgow, Scotland). Isotope ratios are reported in delta notation (δ, in ‰) relative to the international standards V-Pee dee belemnite (carbon) [33] and air (nitrogen) [34]. Analytical error was calculated by running three internal laboratory standards for every ten unknown samples, and four USGS40 isotopic reference samples per plate, to assure good matching of results and allowing any instrument drift to be corrected. Measurement precision of both δ^15^N and δ^13^C was estimated to be ≤ 0.2‰.

### Data and statistical analyses

The infection parameters (prevalence, intensity and mean intensity) were calculated as per Bush et al. [35]. All data were checked for normality and homogeneity of variances. When assumptions were not met, the data were log_10_ or log_10_ (x + 1) transformed. General linear models (GLM’s) were applied to test differences in prevalence and intensity of nematode species between regions. In the cases where the models were significant, Tukey’s HSD *post-hoc* test was used to determine significant differences and grouping using pairwise comparisons. Regression analyses were performed to explore the relationships between (i) nematode intensity in the vent and body (viscera and musculature), vent and viscera, and vent and muscle; and (ii) nematode larvae per gram in the vent and body (viscera and musculature), vent and viscera, and vent and muscle. A full exploration of best line fits to the data using linear, quadratic, and cubic terms was performed on (i) the whole population; and (ii) each separate coastal population for each regression. The curve fitting effectiveness of the different models was assessed using the standard error of the regression (S) and *R*^2^ values. A MANOVA was run to assess overall differences between the combination of δ^13^C and δ^15^N values from different coastal populations of Atlantic salmon. One-way ANOVAs were conducted for δ^13^C and δ^15^N values to identify differences between the coastal populations. All statistical analyses were carried out using Minitab 17 Statistical Software (Minitab Ltd, Coventry, UK).

## Results

Results of the scale readings confirmed that all 117 Atlantic salmon sampled during this study were returning 1-Sea-Winter (1SW) fish. In total, 36,563 Ascaridoidea larvae were collected from 117 Atlantic salmon. These comprised 495 *Hysterothylacium aduncum* (Rudolphi, 1802), 24 *Pseudoterranova decipiens* (Krabbe, 1878) (*sensu lato*) and 30,406 larvae belonging to the genus *Anisakis*. Prevalence and intensity parameters of all three anisakid nematodes recovered in Atlantic salmon are summarised in Table 1 and Table 2. Enzymatic digestion of whole Atlantic salmon carcasses revealed that only 1–7 anisakid larvae (mean ± SD: 3.3 ± 1.8) were missed during the previous fish dissection and examination. As the infestation site of larvae could not be determined, these were not included in further analyses.

**Table 1:** Infection parameters of parasitic nematodes from Atlantic salmon in Scotland

Sampling region	*Anisakis simplex* (*s.l.*)		*Hysterothylacium aduncum*		*Pseudoterranova decipiens* (*s.l.*)	
	Prevalence (%)	MI ± SD	Prevalence (%)	MI ± SD	Prevalence (%)	MI ± SD
East	100	202.9 ± 179.0	59.6	7.9 ± 14.1	17.5	1.4 ± 0.7
West	100	273.8 ± 205.0	73.5	4.6 ± 4.2	14.7	1.6 ± 0.7
North	100	366.7 ± 184.3	73.1	5.9 ± 3.6	7.7	1.0 ± 0.3
Total	100	259.9 ± 197.3	66.7	6.4 ± 10.2	14.5	1.4 ± 0.6

*Abbreviation*: MI, mean intensity; SD, standard deviation

**Table 2:** Historical comparative data for *Anisakis simplex* (*s.l.*) mean intensity and ranges in 1-sea-winter salmon

Sampling region (Year)	*n*	Region of infection				Reference
		Viscera	Musculature	Per fish	Vent	
North-West Atlantic Ocean (1966–1968)	140	2–13	2–9	na	na	[41]
West Greenland (1968)	70	5.3	3.6	na	na	[42]
Montrose, River Spey, Armadale, North and North-East Scotland (2008–2009)	5	na	39.4	na	na	[43]
Strathy Point, North coast Scotland (2009)	10	26.8 ± 12.5^a^	5.1 ± 4.4^a^	63.6 ± 31.9^a^	31.7 ± 21.3^a^	[5]
River Drammenselva, South-East Norway (2009)	17	54.5 ± 66.0^a^	22.8 ± 15.2^a^	89.6 ± 81.5^a^	12 .4 ± 20.8^a^	[9]
Armadale, Usan, and Sandyhills, North, East and West coasts of Scotland (2015)	117	118.2 ± 114.2^a^	40.1 ± 43.4^a^	259.9 ± 197.3^a^	101.6 ± 73.0^a^	This study

^a^Standard deviation

*Abbreviations*: n, number of fish sampled; na, not available

### *Anisakis* species identification

All 30,406 *Anisakis* larvae were morphologically assigned to *A. simplex*, larval type I [23], which corresponds to species within the *A. simplex* (*s.l.*) complex [36]. Pairwise distance calculations based on Kimuraʼs 2-parameter model [31] on the ITS sequence dataset excluding gaps and missing data resulted in no divergence between the four *Anisakis* isolates obtained in this study (0.0%) and *A. simplex* (*s.s.*) sequence (AY826723). Low divergence between the four *Anisakis* isolates studied here was also seen for *A. pegreffii* (0.1%), *A. berlandi* (0.1%) and the *Anisakis* sp. (0.1%) isolated from the northern right whale dolphin, *Lissodelphis borealis* (Peale) (AY821740). The possibility remains that other *Anisakis* spp. sharing the same larval morphotype type I might be present in the samples. Therefore, even though *Anisakis* larvae (*n* = 4) used for molecular identification corresponded to *Anisakis simplex* (*s.s.*), we will refer to the morphotype found in this study as *A. simplex* (*s.l.*).

### Ascaridoid intensities in Atlantic salmon

Ascaridoid intensity per fish showed a significant positive relationship with both FL (*F*_(1, 115)_ = 4.56, *P *< 0.05) and TFW (*F*_(1, 115)_ = 5.49, *P *< 0.05) of Atlantic salmon. Average *A. simplex* (*s.l.*) intensity per fish (musculature, viscera and vent combined) was recorded at 259.9 ± 197.3 from sampled Atlantic salmon during the present study. *Anisakis simplex* (*s.l.*) intensities in the muscle and viscera were recorded at 40.1 ± 43.4 and 118.2 ± 114.3 respectively. Both *H. aduncum* and *P. decipiens* (*s.l.*) were solely recovered from visceral tissues with average intensities of 4.2 ± 10.2 and 0.2 ± 0.6, respectively (Additional file 1: Table S1). Regional differences in *A. simplex* (*s.l.*) intensity were recorded (*F*_(2, 114)_ = 6.91, *P *< 0.001), with significantly higher intensities of *A. simplex* (*s.l.*) observed in Atlantic salmon from the North coast of Scotland (366.7 ± 184.3), compared to those obtained from the East coast (202.9 ± 179.0) (Tukey HSD test, *P *< 0.001). Mean intensity of *H. aduncum* showed no significant differences between regions (*F*_(2, 114)_ = 0.13, *P* = 0.875). *Pseudoterranova decipiens* (*s.l.*) mean intensity was low across East (1.4 ± 0.7), West (1.6 ± 0.7) and North (1 ± 0.3) sample sites.

### The relationship between *A. simplex* (*s.l.*) intensity in the viscera, muscle, and vent region of Atlantic salmon

**Fig. 2:**
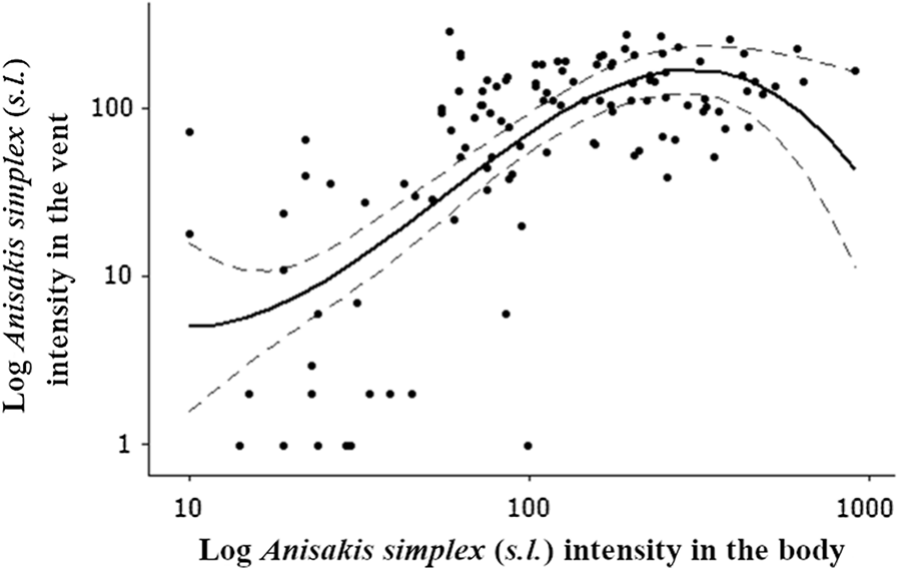
Relationship between *Anisakis simplex* (*s.l.*) intensity in the vent and the body of Atlantic salmon. Body is comprised of viscera and musculature portions of Atlantic salmon. Total sample size was *n* = 117. The area between the dotted grey lines represents the 95% confidence interval for the fitted log cubic polynomial curve

A significant positive relationship was identified between intensity of *A. simplex* (*s.l.*) found exclusively in the vent, and in the musculature and viscera of Atlantic salmon (log cubic, *R*^2^ = 57%, *F*_(1, 115)_ = 5.18, *P *< 0.05) (Fig. 2). When segregated into sample sites, a significant positive relationship was observed in the East (log cubic, *R*^2^ = 62.7%, *F*_(1, 55)_ = 6.75, *P *< 0.05), but not in the North (*F*_(1, 24)_ = 1.63, *P* = 0.215) or West (*F*_(1, 32)_ = 0.90, *P* = 0.350) coast sample sites. No significant relationships were observed between *A. simplex* (*s.l.*) intensities in the vent and muscle (log cubic, *R*^2^ = 28.3%, *F*_(1, 115)_ = 0.71, *P* = 0.400), and vent and viscera (log cubic, *R*^2^ = 62.9%, *F*_(1, 115)_ = 1.28, *P* = 0.261).

Analysis of the relationship of *A. simplex* (*s.l.*) intensity between the viscera, muscle, and vent region of Atlantic salmon was also carried out using *A. simplex* (*s.l.*) larvae per gram of tissue weight (Additional file 2: Table S2). Significant positive relationships of *A. simplex* (*s.l.*) larvae per gram of tissue weight were recorded between the body (viscera and musculature), and the vent (log cubic, *R*^2^ = 38.7%, *F*_(1, 115)_ = 11.42, *P *< 0.001), and the vent and viscera (log cubic, *R*^2^ = 46.8%, *F*_(1, 115)_ = 9.61, *P *< 0.05). There was, however, no significant relationship between larvae per gram in the vent and muscle (log cubic, *R*^2^ = 19%, *F*_(1, 115)_ = 1.71, *P* = 0.194).

### Assessments of feeding ground and dietary composition of Atlantic salmon from different geographical regions using stable isotope analysis

**Fig. 3:**
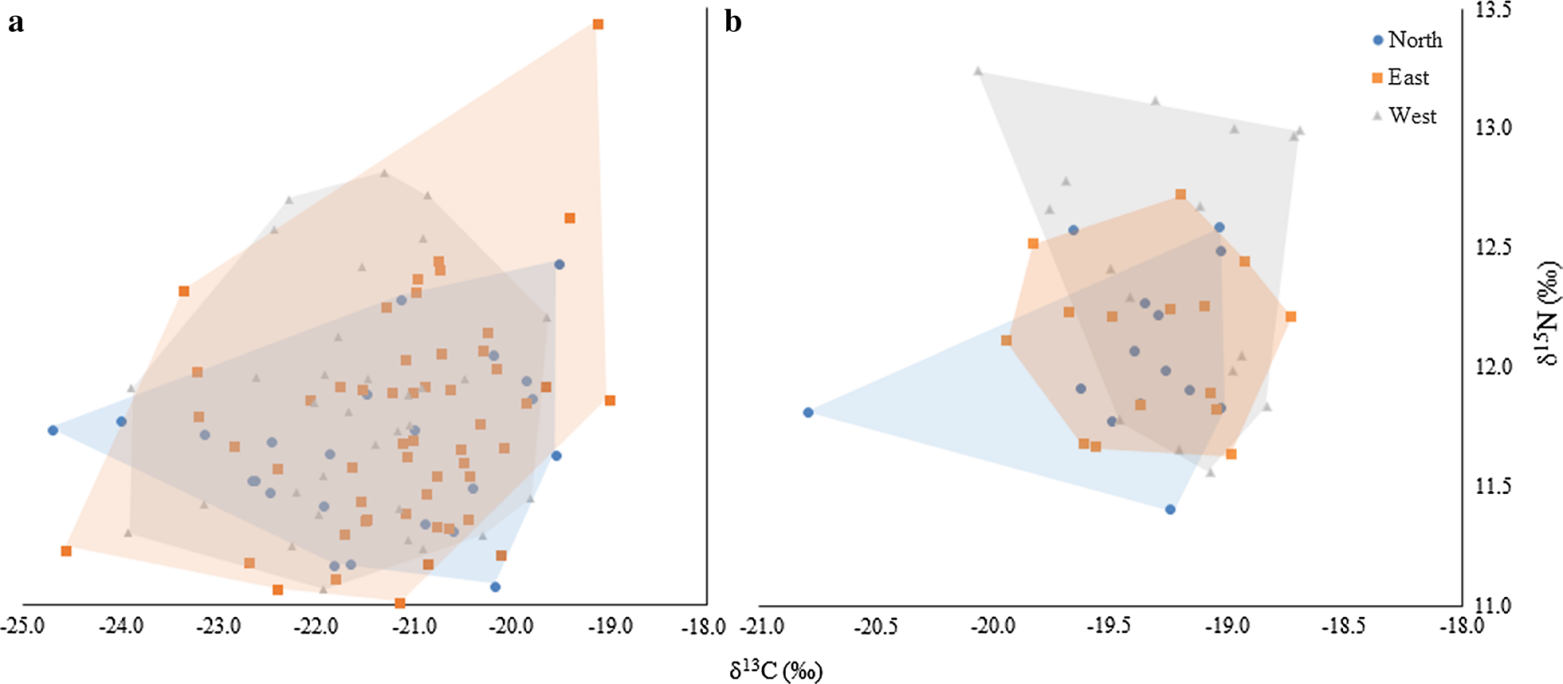
Convex hulls encompassing δ^13^C and δ^15^N signatures of dorsal muscle tissue from Atlantic salmon. Pre-lipid- (**a**) and post-lipid-treated (**b**) Atlantic salmon muscle samples, from the East (*n* = 56), West (*n* = 35) and North (*n* = 25) coasts of Scotland

Details of average δ^13^C and δ^15^N signatures and ranges obtained from pre- and post- lipid dorsal muscle tissue are summarized in Additional file 3: Table S3. No significant differences in δ^13^C and δ^15^N values from dorsal muscle tissue pre-lipid removal were observed between regional sampling sites. (*F*_(2, 115)_ = 1.57, *P* = 0.182, Wilk’s Λ = 0.946) (Fig. 3). Further analysis using one-way ANOVAs similarly found no differences in δ^13^C (*F*_(2, 115)_ = 1.86, *P* = 0.160), and δ^15^N (*F*_(2, 115)_ = 1.15, *P* = 0.32) values between populations. There was also no significant relationship between δ^15^N (cubic, *R*^2^ = 1.3%, *F*_(2, 115)_ = 0.99, *P* = 0.322) and δ^13^C (cubic, *R*^2^ = 3.8%, *F*_(2, 115)_ = 1.71, *P* = 0.193) values of Atlantic salmon and *A. simplex* (*s.l.*) intensity. Following lipid removal, δ^13^C shifts of − 2.15‰, − 2.40‰ and − 1.86‰ were recorded in North, West and East sampling sites, respectively. Lipid removal substantially reduced the range of stable isotope values observed, most notably in the eastern population and to a lesser extent the western. Despite this, it did not result in any change in the results of the analysis; no significant differences between sites were observed.

## Discussion

The ‘novel’ infection of the vent region of Atlantic salmon by *A. simplex* (*s.s.*) [5], and the emergence of RVS, has been suggested to be a result of increasing *A. simplex* (*s.s.*) intensities in the North Atlantic [9]. In the present study we provide new data on *A. simplex* (*s.l.*), *H. aduncum* and *P. decipiens* (*s.l.*) intensities in 1-sea-winter Atlantic salmon sampled in coastal waters of Scotland, which are likely to have potential implications for the angling industry and public health. Furthermore, we assessed the relationship between *A. simplex* (*s.l.*) intensity and distribution within Atlantic salmon tissues, as well as potential differences in dietary composition or feeding ground from different geographical regions.

It had been previously hypothesized that the nematodes present within the ‘novel’ infection site of the vent region of wild Atlantic salmon may represent a different anisakid species from those found in the viscera and body cavity [5]. In the present study, molecular analysis of the ITS sequences of four *Anisakis* isolates taken from the body cavity and vent exhibited very low sequence divergence in comparison to *A. simplex* (*s.s.*) (GenBank: AY826723). These results corroborate with previous studies, which have identified only *A. simplex* (*s.s.*) larvae from Atlantic salmon exhibiting RVS using ITS sequences and restriction fragment length polymorphism (RFLP) patterns [5], and the partial mitochondrial cytochrome *c* oxidase subunit 2 (*cox*2) gene [6].

In comparison to *P. decipiens* (*s.l.*) and *H. aduncum*, much higher prevalence and intensities of *A. simplex* (*s.l.*) were recorded in 1-sea winter Atlantic salmon. The significant difference in prevalence between *P. decipiens* (*s.l.*) and *A. simplex* (*s.l.*) can partially be explained by differences in life histories. Second-stage larvae of *A*. *simplex* (*s.l.*) possess a cocoon-like cuticle which increases buoyancy of third-stage larvae enabling migration within the water column [37]. However, newly hatched *P. decipiens* (*s.l.*) larvae are still ensheathed in the cuticle of the previous second larval stage (L2), attached to the substrate caudally [37]. Larvae are ingested by benthic crustaceans (e.g. amphipods, gammarids and isopods), and transmitted to a large variety of benthic macroinvertebrates acting as second intermediate hosts [38]. As a result, species within the *P. decipiens* (*s.l.*) complex in the northern hemisphere (*Pseudoterranova krabbei* Paggi, Mattiucci, Gibson, Berland, Nascetti, Cianchi & Bullini, 2000, *Pseudoterranova bulbosa* (Cobb, 1888) and *Pseudoterranova decipiens* (*s.s.*)) have a predominantly benthic life-cycle [37, 39]. The larval stage of *P. bulbosa* for example, occurs in benthic fishes, mostly the flatfishes *Hippoglossoides platessoides* (Fabricius), *Reinhardtius hippoglossoides* (Walbaum), and *Hippoglossus hippoglossus* (L.), the cottid *Myoxocephalus quadricornis* (L.), and rarely in the benthopelagic *Gadus macrocephalus* Tilesius. In contrast, *P. krabbei* and *P. decipiens* (*s.s.*) use benthopelagic gadoid fish species as intermediate hosts [40].

As Atlantic salmon spend over 83% of their time feeding within the upper 10 meters of the water column [41], transmission of *A. simplex* (*s.l.*) therefore, is much more likely. Additionally, hydrographic conditions such as fronts play a pivotal role in the life-cycle of *H. aduncum* [42]. Stratified waters can result in increased abundances of suitable hosts (hyperiids) for *H. aduncum* in the North Sea [42]. The availability of suitable intermediate and final hosts of *H. aduncum* in these areas increases the likelihood of their successful transmission [40]. Therefore, the absence of stratified waters and suitable hyperiid hosts in the feeding grounds of Atlantic salmon will have reduced *H. aduncum* transmission. Historical comparisons of *A. simplex* (*s.l.*) intensities in 1-sea winter Atlantic salmon between studies carried out in the late 1960’s, 1970’s [43, 44] to those in 2008 and 2009 [5, 9, 45] revealed a ten-fold increase in North Atlantic and North Sea populations of Atlantic salmon during the intervening 40-year period [9]. In comparison to a similar study of Atlantic salmon in Scottish coastal waters in 2009 (2.19 ± 0.53 kg) [3], the present study demonstrates that eight, three and four-fold increases in *A. simplex* (*s.l.*) intensity have occurred respectively in the musculature, viscera, and per fish. Our results indicate that the trend of increasing *A. simplex* (*s.l.*) intensity in Atlantic salmon since the 1970’s persists (Table 2). Prior to the emergence of RVS in 2005, the ‘hyper-infestation’ of the vent region by *A. simplex* (*s.l.*) had not been recorded in Atlantic salmon or any other fish hosts of anisakid larvae [5]. Senos et al. [9] demonstrated the presence of significant positive relationships between *A. simplex* (*s.s.*) larvae per fish and the number of larvae in the viscera, the musculature (including and excluding the vent) and the vent. A significant positive correlation between the number of larvae in the musculature and vent was also observed. In the present study, we document significant positive relationships between *A. simplex* (*s.l.*) intensities recorded in the body (viscera and musculature) and the vent (Fig. 2). Furthermore, significant positive relationships of *A. simplex* (*s.l.*) larvae per gram of tissue weight were recorded between the body (viscera and musculature) and vent, and the vent and viscera. Moreover, this analysis potentially represents an underestimation of these relationships, as the removal of larval weight from the measured vent weight would result in larger values of larvae/g. These results further support the hypothesis of Senos et al. [9], that the presence of *A. simplex* (*s.s.*) within the ‘novel’ infection site of the vent region [5] is dependent on the intensities of *A. simplex* (*s.s.*) in other body parts of the fish.

**Fig. 4:**
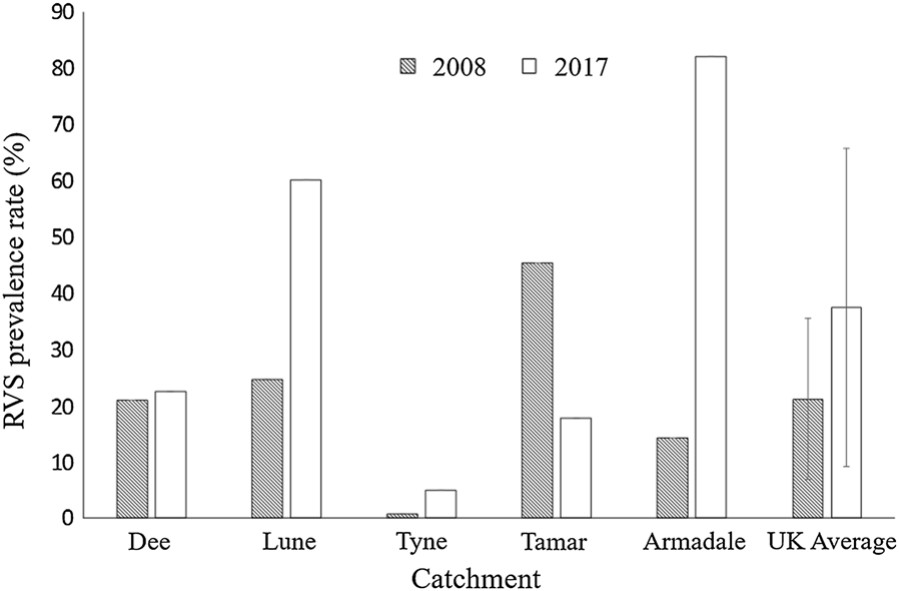
Red Vent Syndrome prevalences (%) recorded in the UK catchment areas in 2008 and 2017. Error bars represent standard deviation

Red Vent Syndrome (RVS) prevalence exhibits high levels of interannual variability (Additional file 4: Table S4). Over the last 10 years, however, there have been increases seen in all monitored catchment areas across the UK except for the River Tamar (Fig. 4). As the presence of *Anisakis* sp. larvae in the vent region has been shown to increase the likelihood of RVS symptoms [8], the significant increase in *A. simplex* (*s.l.*) intensity over the last 50 years is likely to have triggered the infection of the vent region, and the emergence and increasing prevalence of RVS in populations of Atlantic salmon. Our study supports this hypothesis, with the observation of significantly higher *A. simplex* (*s.l.*) intensities in Atlantic salmon captured at netting stations at Armadale, off the North coast of Scotland, where 82% of Atlantic salmon were recorded exhibiting RVS symptoms in 2017 [46].

The substantial increase in *A. simplex* (*s.l.*) intensity in Atlantic salmon over the last 50 years, and differences between geographical regions, are likely due to several biotic and abiotic factors [10]. As Atlantic salmon are opportunistic feeders [16], changes in the availability and distribution of common dietary inputs are likely to cause a change in the dietary composition of wild Atlantic salmon. In recent years, changes in the migratory behaviour of common dietary inputs of wild Atlantic salmon, e.g. Atlantic herring (*Clupea harengus* L.) and Icelandic capelin (*Mallotus villosus* (Müller)), have been concurrent with observations of salmon populations outside the primary feeding grounds (e.g. the Norwegian Sea) of European-originating Atlantic salmon [47]. More specifically, Icelandic capelin has been observed migrating further northwest than previously recorded [47], and Atlantic herring has been recorded migrating to feeding grounds off the east of Iceland, and further northwest towards Greenland [47]. Similar changes in a trophic web in the Gulf of St. Lawrence, Canada [48], where a shift in abundance of prey and an increase in paratenic hosts of *A. simplex* (*s.s.*), have been attributed to significantly higher anisakid infections in Atlantic salmon [8] and Greenland cod (*G. macrocephalus*) [17].

In the present study, δ^13^C or δ^15^N values of Atlantic salmon from different coastal regions of Scotland showed no significant differences. The results of the stable isotope analysis (SIA) suggest that feeding locations and the dietary composition of Atlantic salmon caught in different coastal regions of Scotland are similar. Both δ^13^C (-24.74 to -19.01‰) and δ^15^N (10.53 to 13.44‰) ranges are consistent with previously published data [49], suggesting that there has been no significant change in feeding behaviour of Atlantic salmon over the last 20 years. A number of δ^13^C signatures however, fell outside of the reported δ^13^C range (-26 to -23‰) commonly observed in particulate organic matter from areas North of the Faroe Islands in the Norwegian Sea [50]. These results may be due to interannual fluctuations in δ^13^C signatures [49] or might indicate some variability in feeding grounds between Atlantic salmon.

Stable isotope analysis has previously identified geographical differences in δ^15^N signatures of 1-sea-winter Atlantic salmon in the north-west Atlantic [49]. The dietary composition of Atlantic salmon in the North-West Atlantic consists of higher proportions of fish [51, 52] compared to their European counterparts, where greater proportions of amphipods, krill, mesopelagic shrimp, and squid from lower trophic levels can comprise their diet [53]. Feeding from a wider trophic niche at lower trophic levels by European 1-sea-winter Atlantic salmon is reflected through increased variability of prevailing δ^15^N signatures [54] and could obscure differences in dietary composition. Investigations using SIA on Multi-Sea-Winter (MSW) salmon, which usually exhibit increased preferential feeding of dietary inputs from higher trophic levels, e.g. capelin and Atlantic herring [54], should be pursued in the future to fully clarify any dietary differences in Atlantic salmon originating from Europe.

In addition to SIA, advancements in the differentiation between *Anisakis* sibling species [2], and the population structure of *A. simplex* (*s.s.*) [55], could be used in future studies to clarify spatial differences in Atlantic salmon migratory route. In recent studies, the presence of *A. pegreffii* has been observed in both Atlantic mackerel (*Scomber scombrus* L.) [56], and Atlantic cod (*Gadus morhua* L.) as far north as the Norwegian Sea [57]. Transmission of *A. pegreffii* between migrating mackerel to gadoids in the North Sea, therefore, is present. Furthermore, analysis of mtDNA *cox*2 sequences of *A. simplex* (*s.s.*) isolated from Atlantic herring has revealed a level of genetic sub-structuring that mirrors the population structure of Atlantic herring in the northeast Atlantic [53]. Further molecular investigation into *Anisakis* sibling species, therefore, would significantly aid the assessment of Atlantic salmon migratory route and feeding behaviour. The substantial increase of *A. simplex* (*s.l.*) abundance in Atlantic salmon presents a potential problem for the angling industry [5], to public health [1], and has potentially detrimental ramifications on the wild Atlantic salmon population. In addition to reducing the quality of edible tissue [5], the eight-fold increase of *A. simplex* (*s.l.*) in the musculature over the past 6 years poses a significant threat to public health through their potential to cause gastric, intestinal, ectopic, gastroallergic anisakiasis [1].

Increasing anisakid intensities in Baltic cod since the 1980’s have been associated with decreasing fish condition [58], and a dome-shaped dependency with fish length that has been interpreted as a sign of increasing natural mortality [59]. Increasing anisakid intensities observed in Atlantic salmon may also result in increasing natural mortality adding further pressure to a species, which has already experienced multi-decadal declines [60]. The ultimate cause of increasing *A. simplex* (*s.l.*) intensities observed in Atlantic salmon remains unclear; however, the presence of definitive hosts in regions e.g. fjords of the Faroe Islands [61] has been attributed to substantial increases in nematode infestations in Atlantic cod around the Faroe Plateau [62]. Furthermore, migrations of cetaceans have been associated with ‘spring rises’ of nematode intensities in saithe (*Pollachius virens* (L.)), cod and golden redfish (*Sebastes norvegicus* (Ascanius)) in coastal waters of central Norway [63]. Likewise, increasing grey seal (*Halichoerus grypus* (Fabricius) populations in coastal waters of Denmark have been associated with increased *Contracaecum osculatum* (Rudolphi, 1802) intensities in Atlantic cod [64].

Significantly higher *A. simplex* (*s.l.*) intensities in Atlantic salmon obtained from Armadale off the Scottish North coast, in comparison to those sampled from netting stations on the East and West coast of Scotland were recorded in the present study. These support the observations of similar studies, which have recorded higher *Anisakis* sp. prevalence and intensity in haddock (*Melanogrammus aeglefinus* (L.)) [65], and Atlantic cod [66] in populations sampled from the Barents Sea. With the relative abundance of both large whales and dolphins highest in the Faroe-Shetland Channel [67], and a diverse cetacean population in the Barents Sea [68], these results further demonstrate the potential significance of regional definitive host abundance in prevailing *A. simplex* (*s.l.*) intensities.

The North Sea, particularly in the southernmost sector has experienced profound changes to its climate [69]. As a result, the expansion of cephalopods and spread of sardines and anchovies have been attributed as a primary driver of the increasing occurrence of Risso’s dolphin (*Grampus griseus* (Cuvier)) and common dolphins (*Delphinus delphis* L.) in the central and north-western North Sea [70]. In the marine mammal communities of north-west Scotland, the occurrence of new warm water species including Fraser’s dolphin (*Lagenodelphis hosei* Fraser) and the pygmy sperm whale (*Kogia breviceps* (de Blainville)) have also been recorded since 1980 [15]. Furthermore, the killer whale (*Orcinus orca* (L.)) has been increasingly recorded within the Faroe-Shetland Channel [67, 70]. As a definitive host of *A. simplex* (*s.s.*), the increasing occurrence of both common dolphin and killer whales within these regions could be a potential driver of shifts in *A. simplex* (*s.s.*) intensities in these regions. Additionally, the occurrence of new warm water cetacean species such as Fraser’s dolphin and the pygmy sperm whale, have the potential to introduce other *Anisakis* species, such as *A. pegreffii*, *A. paggiae*, *A. brevispiculata* and *A. physeteris* associated with these definitive hosts, into the region further increasing *Anisakis* spp. numbers.

## Conclusions

Overall our results support the hypothesis of Senos et al. [9] that increases of *A. simplex* (*s.l.*) intensity over the last 50 years have played a significant role in the infection of the vent region [5] and the emergence of RVS in populations of Atlantic salmon. The increasing intensity of *A. simplex* (*s.l.*) in wild Atlantic salmon has the potential to cause significant negative impacts on fish condition, resulting in further increase in mortality rates in wild Atlantic salmon populations that have already suffered multi-decadal declines. As members of the *A. simplex* (*s.l.*) species complex, the known zoonotic potential of *A. simplex* (*s.s.*) and *A. pegreffii* poses risks to consumers [1], and has subsequently prompted the UK Food Standards Agency to issue cautionary advice for the consumption of Atlantic salmon in the UK. Further exploration of the changes in marine mammal communities and their relationship with increasing *A. simplex* (*s.l.*) intensity in the North Sea and north-east Atlantic is required and should be generated in future studies.

## Supplementary information

**Additional file 1: Table S1**. Mean intensity (± SD) of ascaridoid nematode species found in different tissues of 1-sea-winter Atlantic salmon.

**Additional file 2: Table S2**. *Anisakis simplex* (*s.l.*) larvae per gram in different body tissues of Atlantic salmon.

**Additional file 3: Table S3**. Stable isotope values (δ^15^N, δ^13^C) of dorsal muscle tissue from Atlantic salmon in Scotland.

**Additional file 4: Table S4.** Red Vent Syndrome prevalence rates observed in the UK between 2005–2017.

## Data Availability

Data supporting the conclusions of this article are included within the article and its additional files. The newly generated sequences were deposited in the GenBank database under the accession numbers MN313576-MN313579. The datasets used and/or analysed during the present study are available from the corresponding author.

## Abbreviations

*cox*2: cytochrome *c* oxidase subunit 2
FL: fork length
GW: gutted weight
ITS: internal transcribed spacer
L3: third-stage larvae
MSW: multi-sea-winter
mtDNA: mitochondrial DNA
MW: muscle weight
rDNA: nuclear ribosomal DNA
RFLP: restriction fragment length polymorphism
RVS: Red Vent Syndrome
SD: standard deviation
SIA: stable isotope analysis
*s.l*: *sensu lato*
*s.s*: *sensu stricto*
SST: sea surface temperatures
SUERC: Scottish Universities Environmental Research Centre
TFW: total fish weight
VTW: visceral tissue weight
VW: vent weight
1SW: 1-sea-winter
