# Supplementary material for: Increasing intensities of Anisakis simplex third-stage larvae (L3) in Atlantic salmon of coastal waters of Scotland
Source: Parasit Vectors. 2020 Feb 12;13:62. doi: 10.1186/s13071-020-3942-5 (PMC7017554; doi:10.1186/s13071-020-3942-5)
Supplement: Supplementary file 1 — Additional file 1: Table S1. Mean intensity (± SD) of ascaridoid nematode species found in different tissues of 1-sea-winter Atlantic salmon. [file 13071_2020_3942_MOESM1_ESM.docx]

| **Site of infestation** | **Species** | | |
| --- | --- | --- | --- |
|  | ***Anisakis simplex* (s.l.)** | ***Hysterothylacium aduncum*** | ***Pseudoterranova decipiens* (s.l.)** |
| Muscle | 40.1 ± 43.4 | N/A | N/A |
| Viscera | 118.2 ± 114.4 | 4.2 ± 10.2 | 0.2 ± 0.6 |
| Vent | 101.6 ± 73.0 | N/A | N/A |

**Additional file 1: Table S1.** Mean intensity (± SD) of ascaridoid nematode species found in different tissues of 1-sea-winter Atlantic salmon.
