# Supplementary material for: Increasing intensities of Anisakis simplex third-stage larvae (L3) in Atlantic salmon of coastal waters of Scotland
Source: Parasit Vectors. 2020 Feb 12;13:62. doi: 10.1186/s13071-020-3942-5 (PMC7017554; doi:10.1186/s13071-020-3942-5)
Supplement: Supplementary file 2 — Additional file 2: Table S2. Anisakis simplex (s.l.) larvae per gram in different body tissues of Atlantic salmon. [file 13071_2020_3942_MOESM2_ESM.docx]

**Additional file 2: Table S2.** *Anisakis simplex* (*s.l.*) larvae per gram in different body tissues of Atlantic salmon.

| **Population** | ***Anisakis simplex* (s.l.) larvae/g (mean ± SD)** | | | |
| --- | --- | --- | --- | --- |
|  | **Muscle** | **Viscera** | **Vent** | **Body (viscera and musculature)** |
| East (n = 57) | 0.05 ± 0.06 | 0.97 ± 1.05 | 10.22 ± 8.05 | 0.08 ± 0.09 |
| West (n = 34) | 0.02 ± 0.03 | 0.76 ± 0.85 | 14.88 ± 9.93 | 0.07 ± 0.07 |
| North (n = 26) | 0.06 ± 0.06 | 1.33 ± 0.86 | 15.68 ± 7.49 | 0.13 ± 0.10 |
| Total (n = 117) | 0.04 ± 0.06 | 0.99 ± 0.97 | 12.86 ± 8.78 | 0.09 ± 0.09 |
