# Supplementary material for: Increasing intensities of Anisakis simplex third-stage larvae (L3) in Atlantic salmon of coastal waters of Scotland
Source: Parasit Vectors. 2020 Feb 12;13:62. doi: 10.1186/s13071-020-3942-5 (PMC7017554; doi:10.1186/s13071-020-3942-5)
Supplement: Supplementary file 3 — Additional file 3: Table S3. Stable isotope values (δ15N, δ13C) of dorsal muscle tissue from Atlantic salmon in Scotland. [file 13071_2020_3942_MOESM3_ESM.docx]

**Additional file 3: Table S3.** Stable isotope values (δ^15^N, δ^13^C) of dorsal muscle tissue from Atlantic salmon in Scotland.

| **Sample Site** | **Pre-lipid Removal** | | | | | | **Post-lipid Removal** | | | | | | |
| --- | --- | --- | --- | --- | --- | --- | --- | --- | --- | --- | --- | --- | --- |
|  | **δ^15^N (‰)** | | **δ^13^C (‰)** | | | **δ^15^N (‰)** | | | | **δ^13^C (‰)** | | |  |
|  | **Mean ± SD** | **Range** | | **Mean ± SD** | **Range** | | **Sub-sample Size** | **Mean ± SD** | **Range** | | **Mean ± SD** | **Range** | |
| East  (n = 56) | 11.70 ± 0.50 | 10.55 - 13.44 | | -21.18 ± 1.10 | -19.01 - -24.57 | | 15 | 12.10 ± 0.33 | 11.64 - 12.73 | | -19.32 ± 0.36 | -18.74 - -19.95 | |
| West  (n = 35) | 11.75 ± 0.53 | 10.82 - 12.81 | | -21.63 ± 1.01 | -19.64 - -23.93 | | 16 | 12.44 ± 0.56 | 13.24 - 11.56 | | -19.23 ± 0.39 | -18.69 - -20.07 | |
| North  (n = 25) | 11.56 ± 0.44 | 10.53 - 12.43 | | -21.56 ± 1.52 | -19.52 - -24.74 | | 14 | 12.05 ± 0.34 | 11.40 - 12.59 | | -19.41 ± 0.45 | -19.03 - -20.79 | |
