# Supplementary material for: Increasing intensities of Anisakis simplex third-stage larvae (L3) in Atlantic salmon of coastal waters of Scotland
Source: Parasit Vectors. 2020 Feb 12;13:62. doi: 10.1186/s13071-020-3942-5 (PMC7017554; doi:10.1186/s13071-020-3942-5)
Supplement: Supplementary file 4 — Additional file 4: Table S4. Red Vent Syndrome prevalence rates observed in the UK between 2005–2017. [file 13071_2020_3942_MOESM4_ESM.docx]

| **Catchment** | **2004** | **2005** | **2006** | **2007** | **2008** | **2009** | **2010** | **2011** | **2012** | **2013** | **2014** | **2015** | **2016** | **2017** |
| --- | --- | --- | --- | --- | --- | --- | --- | --- | --- | --- | --- | --- | --- | --- |
| Dee | 0.4 ^(a)^ | 3.2 ^(a)^ | 9.2 ^(a)^ | 29.9 ^(a)^ | 20.9 ^(a)^ | 28.2 ^(a)^ | 23.7 ^(a)^ | 10.9 ^(a)^ | 13.2 ^(a)^ | 20.5 ^(a)^ | 25.3 ^(a)^ | 24.4 ^(a)^ | 21.7 ^(a)^ | 22.5 ^(a)^ |
| Lune | - | 0 ^(a)^ | 1.4 ^(a)^ | 23.1 ^(a)^ | 24.7 ^(a)^ | 21.2 ^(a)^ | 18.8 ^(a)^ | 16.3 ^(a)^ | 0 ^(a)^ | 41.6 ^(a)^ | 9.5 ^(a)^ | 13.6 ^(a)^ | 19 ^(a)^ | 60.2 ^(a)^ |
| Tyne | - | - | - | 1.4 ^(a)^ | 0.8 ^(a)^ | 3.4 ^(a)^ | 5.3 ^(a)^ | 3.8 ^(a)^ | 5.2 ^(a)^ | 10.1 ^(a)^ | 7.5 ^(a)^ | 10.3 ^(a)^ | 3.5 ^(a)^ | 4.9 ^(a)^ |
| Tamar | - | - | - | 60.2 ^(a)^ | 45.3 ^(a)^ | 41.5 ^(a)^ | 57.1 ^(a)^ | 45.6 ^(a)^ | 26.1 ^(a)^ | 44.5 ^(a)^ | - | 35.5 ^(a)^ | 24.6 ^(a)^ | 17.7 ^(a)^ |
| Caldew | - | - | - | 5.3 ^(a)^ | 0.3 ^(a)^ | 10.2 ^(a)^ | 5.1 ^(a)^ | 6.4 ^(a)^ | 6.1 ^(a)^ | 0.8 ^(a)^ | - | - | - | - |
| England & Wales Average | 0.4 ^(a)^ | 1.6 ^(a)^ | 5.3 ^(a)^ | 24.0 ^(a)^ | 17.7 ^(a)^ | 20.9 ^(a)^ | 22 ^(a)^ | 16.6 ^(a)^ | 10.1 ^(a)^ | 23.5 ^(a)^ | 14.1 ^(a)^ | 21.0 ^(a)^ | 17.2 ^(a)^ | 37.5 ^(a)^ |
| N. Esk | - | - | - | - | 14.4 ^(b)^ | 30.9 ^(c)^ | 26.8 ^(c)^ | - | - | - | - | - | - | - |
| Oykel | - | - | - | - | 6.6 ^(b)^ | 85.4 ^(c)^ | 50 ^(c)^ | - | - | - | - | - | - | - |
| Solway/Annan | - | - | - | - | 17.9 ^(b)^ | 17.9 ^(c)^ | - | - | - | - | - | - | - | - |
| Tweed | - | - | - | - | 12.9 ^(b)^ | 20.7 ^(c)^ | - | 30.6 ^(c)^ | - | - | - | - | - | - |
| Melvich | - | - | - | - | 17.5 ^(b)^ | 69.3 ^(c)^ | 23.2 ^(c)^ | - | - | - | - | - | - | - |
| Armadale | - | - | - | - | 14.3 ^(b)^ | 71.2 ^(c)^ | 30.7 ^(c)^ | - | - | - | - | - | - | 82 ^(d)^ |
| Scotland Average | - | - | - | - | 12.9 ^(b)^ | 43.5 ^(c)^ | - | - | - | - | - | - | - | - |

**Additional file 4: Table S4.** Red Vent Syndrome prevalence rates observed in the UK between 2005–2017.

Superscript indicates source of data: (a) International Council for the Exploration of the Sea (ICES). Preliminary assessment on salmon stocks and fisheries in England and Wales. 2017. <https://assets.publishing.service.gov.uk/government/uploads/system/uploads/attachment_data/file/808448/SalmonReport-2018-background_final.pdf>. Accessed 6 Mar 2019; (b) Pert CC, Noguera PA, Bruno DW. Scottish Red Vent Syndrome survey 2008. Internal Report No 07/09. Aberdeen: Marine Scotland Science; 2009. <https://www2.gov.scot/Uploads/Documents/Int0709c.pdf>. Accessed 6 Jan 2017; (c) Pert CC, Personal communication, 2015; (d) [46].
